# An Updated Review of the Genus *Humulus*: A Valuable Source of Bioactive Compounds for Health and Disease Prevention

**DOI:** 10.3390/plants11243434

**Published:** 2022-12-08

**Authors:** Katya Carbone, Fabio Gervasi

**Affiliations:** CREA—Research Centre for Olive, Fruit and Citrus Crops, Via di Fioranello 52, 00134 Rome, Italy

**Keywords:** *Humulus*, hops, disease prevention, phytoestrogens, bioactive compounds, clinical studies

## Abstract

The medicinal potential of hop (*Humulus lupulus* L.) is widely cited in ancient literature and is also allowed in several official pharmacopoeias for the treatment of a variety of ailments, mainly related to anxiety states. This is due to the plethora of phytoconstituents (e.g., bitter acids, polyphenols, prenyl flavonoids) present in the female inflorescences, commonly known as cones or strobili, endowed with anti-inflammatory, antioxidant, antimicrobial, and phytoestrogen activities. Hop has recently attracted the interest of the scientific community due to the presence of xanthohumol, whose strong anti-cancer activity against various types of cancer cells has been well documented, and for the presence of 8-prenyl naringenin, the most potent known phytoestrogen. Studies in the literature have also shown that hop compounds can hinder numerous signalling pathways, including ERK1/2 phosphorylation, regulation of AP-1 activity, PI3K-Akt, and nuclear factor NF-κB, which are the main targets of the antiproliferative action of bitter acids and prenylflavonoids. In light of these considerations, the aim of this review was to provide an up-to-date overview of the main biologically active compounds found in hops, as well as their *in vitro* and *in vivo* applications for human health and disease prevention. To this end, a quantitative literature analysis approach was used, using VOSviewer software to extract and process Scopus bibliometric data. In addition, data on the pharmacokinetics of bioactive hop compounds and clinical studies in the literature were analysed. To make the information more complete, studies on the beneficial properties of the other two species belonging to the genus *Humulus, H. japonicus* and *H. yunnanensis*, were also reviewed for the first time.

## 1. Origins of Hops and Early Official Uses

**Figure 1:**
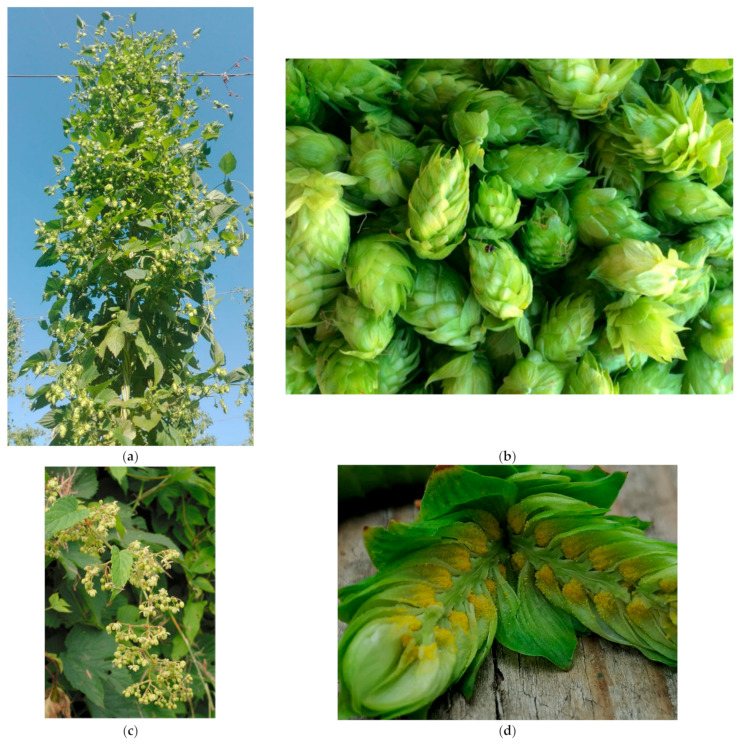
(**a**) Hop plant; (**b**) hop cones; (**c**) hop male inflorescences; (**d**) cross section of a hop cone containing lupulin.

Among the medicinal plants with interesting biological properties is certainly the hop (*Humulus lupulus* L., Figure 1a), known to most for its use in the brewing industry, but recently also finding increasing use in the medicinal sector (Table 1). Its strong therapeutic potential is due to the presence, especially in the cones, of a wide range of bioactive molecules, mainly secondary metabolites, some of which characterise the plant itself, such as bitter acids and specific prenylflavonoids such as xanthohumol (XN) or 8-prenylnaringenin (8-PN) [1].

To date, the origin of hops is uncertain, but the presence of the three species of the genus *Humulus* (*H. lupulus, H. yunnanensis*, and *H. japonicus*) in China has suggested that the first hop species appeared in Asia and spread from there eastwards to North America and westwards to Europe [1,57]. Hop pollen has been discovered in archaeological sites in England dating back to 3000 B.C. (Stone Age) and it is known that since ancient Egyptian times, hops were used as a medicinal herb [1,58,59]. During Roman times, hops gained importance for their use in the treatment of liver diseases, digestive disorders, and as a blood purifier. When the Romans occupied Britain, they began to regard hops as a delicacy, using them to make infusions and adding them to cereal fermentations, along with other ingredients used at the time [1]. As tastes and customs changed, all the ingredients used in the fermentation process fell into disuse except for hops, which remained and became a key element in the preparation of beer. The oldest report on the medicinal uses of hops can be found in a book dating back to the Middle Ages, specifically to the 11th century, in which the Arabian physician Mesue described the anti-inflammatory properties of this perennial herb [60]. In the 13th century, the Arabian botanist Ibn al-Baytar highlighted the soothing properties of hops [61]. Between 1300 and 1600, there was a widespread use of hops as a remedy for fevers, spleen disorders, as a diuretic, and for liver purging [60]. In North America, various indigenous tribes used hops as a remedy for various ailments [1]. The Delaware, for example, used it for earache and toothache; the Cherokee used it against sleeping disorders [62]; the Navajo against coughs and colds; and the Dakota used hop infusions as a cure for intestinal disorders and wound healing [63]. Hops were also used in Ayurvedic medicine, a traditional medicine used in India since ancient times and still widespread in the subcontinent today [64,65]. Although there was presumably no exchange of knowledge on the action of hops, the uses of the plant were common between the various continents. In this regard, one can consider this parallel use as indirect proof of the effectiveness of hops in medical applications. In 1820, the physician Ansel W. Ives examined the resin glands of hops and first proposed the name “lupulin”, also pointing out its peculiar sedative properties [66]. The name “*Humulus lupulus”* was given by Carl Linnaeus. The term *Humulus* is thought to derive from the Latin *humus* (earth), alluding to the plant’s flexible stems resting without a support on the ground; *lupulus*, on the other hand, may originate from the Latin word ”*lupus”*, justified, according to some, on the basis of a reference by Pliny the Elder (23–79 AD), who referred to the plant as the “wolf of the willows” [1]. In contrast, the common name “hop/hops” seems to be of Anglo-Saxon derivation (hoppan: to climb; [67])

## 2. Botanical Classification

Hop is an herbaceous perennial plant belonging to the class Magnoliopsida, subclass Hamamelididae, order Urticales, family Cannabaceae, genus *Humulus*. Two other species belong to the genus: *H. japonicus* and *H*. *yunnanensis* [68]. All *Humulus* species are climbing plants, with hooked trichomes for grasping and wrapping around available support structures.

## 3. Humulus Japonicus/Humulus Scandens

*Humulus japonicus* (HJ) is native to Japan, Taiwan, and China [68]. It is also known with the synonym *Humulus scandens*; however, the European and Mediterranean Plant Protection Organization, www.eppo.int (accessed on 1 September 2022), lists *H. scandens* as the accepted name.

It is a dioecious species, typically annual, although it may survive for more than one season. *H. scandens* is a highly resilient species, with good vigour and able to grow between 500 and 1500 m asl, adapting to the soil texture and different climatic soil conditions; it is widely distributed in northern temperate and subtropical zones, especially in China, Korea, and Japan. The female inflorescences are like hop cones but, unlike the latter, they are not used for fermentation due to the scarce presence of resinous glands within them. It is used as an ornamental plant, although its strong climbing trichomes make it difficult to handle [68].

Yu et al. (2007) highlighted important biological properties of this plant [69]. Traditionally, it was used to treat hypertension, pulmonary disease, and leprosy [70]. *H. scandens* is rich in flavonoids, which are distributed among different plant tissues; among them vitexin, luteolin–7–O–β–D-glucoside, apigenin–7–O–β–D-glucoside, and cosmosiin have been separated and identified [71,72]. The authors also demonstrated that the total flavonoids of HJ, together with luteolin glycoside, show a significant inhibition effect on the human hepatocellular carcinoma cell line [72]. Recently, Zhang et al. (2021) identified 15 different compounds in the n-butanol extract of *H. scandens,* highlighting among others the presence of astragalin also known in *H. lupulus* [73]. The authors also pointed out the significant anti-inflammatory effects and the absence of signs of behavioural or neurological toxicity in mice treated orally with *H. scandens* extract up to 2000 mg/kg body weight: in addition, extracts in chloroform and n-butanol improved superoxide dismutase (SOD) and catalase (CAT) activity in mice. However, the biological properties seemed closely related to the type of solvent used and thus to the phytochemical profile of the extract tested.

Park et al. (2021) also demonstrated the beneficial potential of *H. scandens* for the treatment of symptoms related to autism spectrum disorder [70]. Moreover, Wang et al. (2022) reported that an ethanolic extract (80% *v*/*v*) of *H. scandens* leaves demonstrated antioxidative and antiapoptotic effects and could be developed as a promising candidate for the prevention and treatment of neurodegenerative diseases related to oxidative stress, such as Parkinson’s. The authors found that the extract significantly reduced reactive oxygen species (ROS) production and increased levels of endogenous antioxidants, inhibiting 6-hydroxydopamine (6-OHDA)-induced activation of the mitochondrial apoptotic pathway in PC12 cells, potentially leading to neuronal cell protection. Chemical analysis of the extract revealed among the most abundant components luteolin-7-O-glucoside and apigenin-7-O-glucoside, molecules known to significantly inhibit the enzymatic activity of monoamine oxidase B, which plays an important role in the catabolism of neuroactive and vasoactive amines in the central nervous system and peripheral tissues (such as dopamine) [74]. The same extract was further tested to assess its anti-inflammatory impact on lipopolysaccharide-stimulated SIM-A9 cells, showing its own and luteolin’s ability to inhibit, among all, the generation of interleukin-1β, tumour necrosis factor-α, nitric oxide, and interleukin-6 [75]. Recently, Kang et al. (2022) demonstrated that a water extract of HJ, when orally administered (500 mg/kg/day for 3 weeks), significantly decreased β amyloid plaque deposition in the cerebral cortex and hippocampus of Alzheimer’s disease (AD) mice models, also protecting against AD-related memory impairments via enhancing the cholinergic system [76]. Feng et al. (2014) investigated the immunosuppressive effects of a methanolic extract of *H. scandens* on T-cell activation both *in vitro*, on splenocytes, and *in vivo*, on mice [77]. The in vitro results demonstrated that the extract possessed significant inhibitory effects on splenocyte proliferation, and its immunosuppressive activities were not due to the toxicity. The extract was also able to suppress T-cell activation *in vivo*, showing no apparent toxicity symptoms or significant changes in body weight at the doses tested (100 and 50 mg/kg).

## 4. Humulus Yunnanensis

*Humulus yunnanensis* is a species endemic to China’s Yunnan province and is also used in brewing and pharmaceuticals [78]. The plant is perennial and, like the others in this genus, dioecious. The species was identified by Ernest Small in 1978 who found that, unlike the previous species, it could grow at lower latitudes. Currently, both few specimens of *H. yunnanensis* and few studies in the literature are available. In 2020, a new variety of *H. yunnanensis* called “Kriya” was patented in the United States for medical uses, whose leaves contain a cannabinoid level of about 15–23 mg/g and inflorescences a cannabinoid level of about 124–142 mg/g [79,80].

However, Appendino et al. (2022), in their review on the presence of cannabidiol in non-cannabis plants, critically argue the alleged fraudulence of the finding, which, according to the authors, is highly dubious in scientific terms, especially given the absence in the plant of the metabolic pathway for phytocannabinoid biosynthesis [81].

## 5. Humulus Lupulus

Hop is a dioecious, perennial plant that produces for about 25–30 years and whose growth can reach 30 cm per day in the peak vegetative period, reaching heights of 7–10 m [82]. The commercially useful part of the plant is the female inflorescences, called strobili or, more commonly, cones (Figure 1b). In contrast, male plants, containing flowers capable of producing pollen (pollinator plant), are kept away from the growing areas to prevent the formation of seeds within the cones because of pollination, thus ensuring a genetically constant product [82]. However, they are essential for the breeding program [1]. Hops flower in the summer: the male inflorescences are panicle-shaped (Figure 1c), while the female inflorescences are ovoid in shape and are formed by a central rachis bearing bracts and bracteoles (small bracts) at each node. During flowering the rachis elongates, the size of the bracts increases, and, in the lower part of the bracts, a large quantity of glandular trichomes containing a yellow resin called lupulin develops (Figure 1d) [1]. Lupulin accounts for 10–30% of the dry weight of strobili, depending on the hop variety. It is rich in α- and β-acids and essential oils. The number and size of glands depend mainly on genetic differences among varieties [83].

### 5.1. Chemical Composition of H. lupulus

To date, more than 1000 compounds have been identified in hops, including essential oils, bittering substances, and prenylflavonoids [84]. The most important active ingredients are contained in the glandular structures (50–80%), the remaining 15–30% in the bracts (Table 2; [1,85]).

#### 5.1.1. Primary Metabolites

The literature gives us little information regarding the primary metabolites of hops. For sugars, the percentages derived from dried hop cones of glucose, fructose, and sucrose have been identified, as reported by MacWilliam [86]. Carbone et al. (2020) firstly reported the identification and quantification of the most concentrated primary metabolites in hop cones, mainly amino acids and sugars, using ^1^H NMR spectral deconvolution [87]. The most abundant sugars were glucose (range: 52–58% *w*/*w*) and fructose (range: 27–32% *w*/*w*), followed by maltose > galactose > xylose~mannose. A few other metabolites, including *myo*-inositol and lactate (carbohydrate metabolism), choline, *o*-phosphocholine and ethanolamine (lipid metabolism) and two signalling molecules; 4-aminobutyrate and trigonelline, were also found in hop cone extracts obtained via ultrasound- and microwave-assisted extractions [87]. Literature studies report that trigonelline can be used as an antidiabetic, antibacterial, antiviral, sedative, anti-migraine, and antitumour agent [88,89].

Among amino acids, asparagine accounts for 75% by weight of the total amino acids detected in green hop extracts [87]. Other amino acids found were proline, alanine, valine, isoleucine, threonine, and leucine [87]. Oostervald et al. (2002) extracted, under acidic conditions, pectin from spent hops, with a yield of 2%, containing 59% polysaccharides. The pectin had a relatively high molecular weight and an intrinsic viscosity comparable to that of commercially available apple and citrus pectin [90].

Recently, Chen et al. (2020) isolated and characterized a novel homogeneous heteropolysaccharide (HLP50-1; 49.13 kDa) obtained from the female flowers of *H. lupulus*, which can promote osteoblast differentiation and mineralisation, making it a candidate for the treatment of osteoporosis [91].

In a recent study, Nezi et al. (2022) reported the percentage distribution of different protein classes found in hop cones from Cascade and Chinook varieties grown in southern Italy [92]. The most abundant was the class related to photosystems (19%), followed by those related to ATP synthase and flavonoid pathways.

#### 5.1.2. Secondary Metabolites

The bioactive secondary metabolites of hops are synthesized mainly in trichomes in the form of the yellow resin, lupulin [39]. Resins are divided into “hard resins” and “soft resins” based on their relative solubility in organic solvents. Substances soluble in hexane, paraffinic hydrocarbons and in petroleum ether belong to the so-called “soft” resins. Substances insoluble in these solvents belong to the “hard” resins [83].

The secondary metabolites of hops can be divided into three major families: bitter acids, polyphenols, and essential oils. In the leaves, due to the presence of a small number of glandular trichomes, prenylated flavonoids, tannins, and glycosylated flavonols can be found [93]. Interest in these metabolites has grown considerably in recent years, not only because of the explosion of the craft beer phenomenon, but also because of their remarkable biological properties of health interest.

##### Bitter Acids

**Figure 2:**
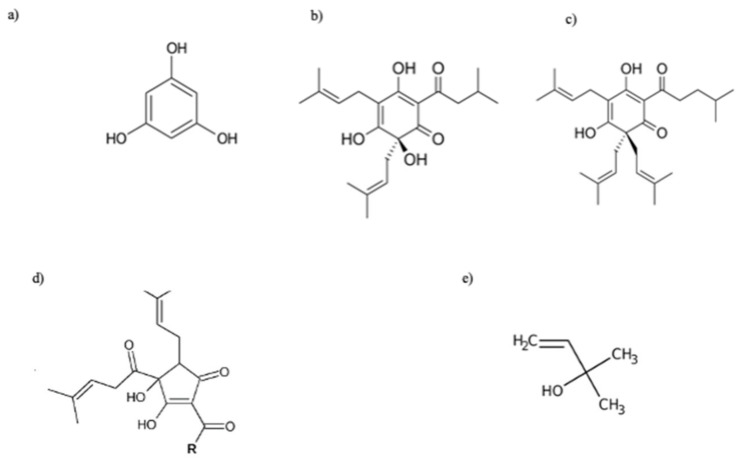
Chemical structure of (**a**) phloroglucinol (benzene-1,3,5-triol); (**b**) humulones; (**c**) lupulones; (**d**) iso-α-acids; (**e**) 2-methyl-3-buten-2-ol.

Hop bitter acids are prenylated derivatives of phloroglucinol (1,3,5-trihydroxybenzene, C_6_H_6_O_3_; Figure 2a), occurring as soft, pale yellow oils or resins soluble in hydrocarbon solvents such as hexane (soft resins). Bitter acids are divided into two main chemical classes: α-acids (humulones; Figure 2b) and β-acids (lupulones; Figure 2c), which differ in the substituent in C6: in β-acids, the hydroxyl group present in α-acids is replaced by a prenyl group. Depending on the acyl side chain, they can be distinguished into n- (isovaleryl moiety), co- (isobutyryl moiety), ad- (2-methylbutyroyl moiety), pre- (isohexanoyl moiety), post- (propanoyl moiety) α- and β-acids. The α-acid portion of the resin is composed of about 15% adhumulone, 20–50% cohumulone, and 20–50% humulone, with the amounts of cohumulone and adhumulone varying by hop variety [94].

During the wort boiling process, at alkaline pH (8–10), humulones undergo isomerization, via an acyloin-type ring contraction, to produce the corresponding iso-α-acids (iso-humulone, iso-cohumulone, and iso-adhumulone; Figure 2d) [95]; these compounds are more bitter and soluble than their precursors, are responsible for the bitterness of beer, and, for this reason, define the commercial value of the hops themselves. The presence of a prenyl group in the chemical structure of β-acids makes them less acidic than α-acids, and even when subjected to the same conditions as α-acids during wort boiling, it does not allow their isomerization. Consequently, they have limited brewing values, also due to a lower solubility in wort and beer than the alpha ones; however, β-acids are critical to the microbiological stability of beer due to their high antiseptic power [96].

Recently, hop bitter acids received attention due to their biological properties, mainly antimicrobial ones [6].

##### Essential Oils

Another commodity class of hop compounds is essential oils (EOs), which are secreted by lupulin glands. To date, more than 1000 different compounds have been identified in hop EO, which are considered “essential” because they give hops their characteristic aroma and help define the taste of beer. On average, dried hops contain 0.5 to 3.0 percent EOs [97], and both their amount and composition depend largely on genetic factors, the age of the plant, characteristics of the growing soil, but also on climatic conditions (temperature, humidity, hours of sunshine) and time of harvest [98]. Their biosynthesis, which takes place in trichomes, occurs more slowly than that of resins; consequently, hop oils are fully developed in the last stage of hop maturation [27].

Chemically, they can be divided into two main subgroups: hydrocarbons and oxygenated compounds. Hydrocarbons (about 50–80% of the total hop oil content) are a group of substances to which very volatile chemical compounds belong. Their solubility in water, wort, and beer is poor; in fact, most of these compounds are lost by evaporation during the boiling process. They in turn can be divided into three groups: i) aliphatic hydrocarbons (e.g., isoprene), ii) monoterpenes (two isoprene units; e.g., myrcene, ocimene, pinene, limonene, and cymene) and iii) sesquiterpenes (three isoprene units; e.g., humulene, caryophyllene, and farnesene) [27]. Among the hydrocarbons, the most important and abundant monoterpene found in hop EO is myrcene, which can account for 30 to 60% of the total oil content in the cone and is primarily responsible for the pungent vegetal odour of fresh hops; during boiling, it also forms other aromatic compounds, particularly fragrant, such as linalool and geraniol. Sesquiterpenes are less volatile than monoterpenes and less prone to oxidation [27]. Humulene is the most abundant sesquiterpene found in hops; it is described as having a delicate, refined, and elegant scent and is mainly found in aromatic hops [99]. Oxygenated compounds (about 30% of total hop oil content) include: aliphatic alcohols (e.g., methyl alcohols, isopentyl alcohols, etc.), terpene alcohols (e.g., linalool, geraniol, etc.), aliphatic ketones, aliphatic aldehydes, terpene aldehydes, and epoxide esters. Because of their higher solubility in water than the hydrocarbon fraction, their impact on beer aroma is high [27]. Sulphur compounds are also present in low concentration in hop EO, but their odour impact is considerable. Among them, the most abundant ones are S-methylthiomethyl thioesters, which are characteristic of hops [27]. Several studies highlighted the healthy properties of hop EOs, ranging from sedative to antimicrobial [100,101].

##### Nonphenolic Pigments

In addition to bitter acids and volatile compounds, nonphenolic pigments (chlorophyll a, chlorophyll b, and carotenoids) are also present in hops [102]. These are an important class of secondary metabolites that can contribute to the prevention/reduction of various degenerative disorders, such as atherosclerosis, osteoporosis, cataracts, neurodegenerative diseases, and oxidative stress [103]. They can also exert an important antioxidant action, particularly against the hydroperoxide radical [93]. Recently, Macchioni et al. (2022) reported that ethanolic extracts from freeze-dried hop leaves exhibited a total chlorophyll content in line with the average content of green tea leaves (1.47 mg/g) [93]. In addition, the supercritical CO_2_ extract from hop pellets (cv. “Ella”) exhibited a total amount of nonphenolic pigments that did not exceed 0.04% of the total mass of the extract. The authors also pointed out that this content was closely related to the applied extraction pressure and could also influence the antioxidant capacity of the extract in the *in vitro* ORAC test [104]. García-Herrera et al. (2013) analysed the carotenoid content in young edible shoots of *H. lupulus*, grown in Spain. The authors reported the presence, over two years of observations, of neoxanthin, the most abundant compound (about 7 μg/g wet weight); lutein; β-carotene; and violaxanthin, the least abundant one (about 3 μg/g wet weight) [105].

To date, to the best of our knowledge, there are no studies related to the potential of nonphenolic hop pigments for human health.

##### Polyphenols

Polyphenols make up to 14% of the total weight of the dried cones and they are present mainly in the bracts [39,106]. In their recent review, Sun et al. (2022) reported the main polyphenolic compounds found in hops [107]. They are divided into two main classes: non-glycosylated and glycosylated polyphenols [60]. The main glycosylated polyphenols found in hop cones are rutin and isoquercitrin (quercetin 3-*O*-b-D-glucopyranoside; [60]). Among the non-glycosylated polyphenols are phenolic acids (i.e., benzoic and cinnamic acid derivatives), coumarins, and flavonoids [60]. According to Biendl (2009), the most representative phenolic class in dried hop cones (10% residual moisture) is flavanols (7%), followed by prenylflavonoids (1.3%), flavonols (0.5%), and phenolic acids (0.05%) [61].

Flavonoids, low-molecular-weight polyphenols consisting of two aromatic rings connected by a heterocycle, are particularly important for human health. Based on the type of heterocycle and the substituents of the benzene rings, flavonoids are divided into: prenylflavonoids, anthocyanidins, flavonols, flavans, flavanones, flavones, isoflavones, and hydrolysable tannins [108]. The hop polyphenolic profile varies, both compositionally and quantitatively, as genotype, agronomic and climatic factors vary depending on the type of extract tested [99].

Santarelli et al. (2022) observed that in hop extracts (cv. ”Cascade”), depending on the extraction process adopted and the solvent used, the most abundant compounds were catechin and xanthohumol, with concentrations varying, depending on the extraction method used, from 1.22 to 2.70 mg/g and from 1.09 to 2.67 mg/g, respectively. In the same study, rutin and chlorogenic acid ranged from 0.612 to 0.877 mg/g, while *p*-hydroxybenzoic, protocatechuic, syringic, and ellagic acids ranged from about 0.262 to 0.654 mg/g. Other polyphenols, such as epigallocatechin, *p*-coumaric acid, caffeic acid, and ferulic acid, were found in lower concentrations and to very different extents depending on the type of extraction applied [109]. Kowalska et al. (2022) analysed pharmaceutical grade herbs of hop cones and found that the total polyphenol content ranged from 3.3 to 4.2%, of which flavonoids accounted for about 0.16 to 0.37% [106].

Not only the cones, but also other plant tissues are important sources of polyphenols. Recently, Chiancone et al. (2022) in their pre-print article evaluated *in vitro* hop seedlings (cv. “Cascade” and “Gianni”) as valuable bio-factories of hop polyphenols, identifying, in the leaves and roots, phenols belonging to different classes (i.e., prenylated chalcone, glycosylated flavonols, flavan-3-ols, and phenolic acids, derived both from benzoic acid and cinnamic acid) [110]. In hop seed extracts, catechin was found as the most abundant compound (13.7 ± 0.5 mg/g extract), followed by (-)-epicatechin (3.9 ± 0.2 mg/g extract) [111]. Furthermore, Maietti et al. (2017) found kaempferol derivatives, such as kaempferol-3-O-(6′-O-malonyl)-glucoside (or galactoside) (770–226 µg/g), kaempferol-3-O-glucoside (491–65 µg/g) and kaempferol-3-O-(6′-O375 malonyl)-neohesperidoside (401–54 µg/g) as the most abundant compounds in different hop shoot samples [112].

In the last 20 years, however, the most important and studied hop phenol class is undoubtedly the prenylflavonoids, as they characterize the phenolic profile of hops. These are distinguishable according to the prenyl group, which shows important biological activities and benefits to human health [52].

##### Hop Prenylflavonoids

Belonging to this group of molecules are two prenylcalcones, xanthohumol (XN) and desmethylxanthohumol (DMX), and three prenylflavanones, isoxanthohumol (IX), 6-prenylnaringenin (6-PN), and 8-prenylnaringenin (8-PN). These compounds differ in the position of the prenyl group, the number of isoprene units, and modifications of the prenyl moiety, such as cyclization and hydroxylation. Recently, these compounds, by virtue of their biological activity and therapeutic potential for human health, particularly their estrogenic effect, have received more attention than the other polyphenols found in the plant [113]. XN, also found in another medicinal plant, the *Sophora flavescens* [114], is the main prenylflavonoid in hops (0.3–1.5% *w*/*w*; [115]) and is a source of IX; in fact, XN, during the boiling phase of beer, isomerizes, thanks to the free hydroxyl group that can participate in the cyclization reaction, producing precisely IX [59]. The isomerization also occurs in the stomach, due to its acidic conditions [116]. Another compound that tends to isomerize is DMX, which, with two free ortho-hydroxyl groups in C10, isomerizes into 6- and 8-PN [101].

As observed for other hop metabolites, the content of prenylated flavonoids in hop cones is related to genetic, environmental, and agronomic factors. Maliar et al. (2017) evaluated the XN and DMX contents of eight hop cultivars, recording XN contents between 0.4 and 0.9% and DMX contents between 0.1 and 0.3%, depending on the genotype analysed [117]. The highest contents of XN (1.0%) were reported for cv. “Taurus”, ”Admiral” and ”Agnus”, while the highest contents of DMX (0.14–0.18%) were reported for cv. ”Magnum”, ”Sládek”, etc.

## 6. Scientometric Analysis on Hop’s Biological Properties for Health

In order to take a snapshot and characterize the literature published over the years (1984–1 September 2022) regarding the health properties of *H. lupulus*, a scientometric analysis was performed by coupling the use of the Scopus online database (www.scopus.com) to the VOS viewer software (v. 1.6.16, 2020).

The Scopus search (string: “Article title, Abstract, Keywords”) using the keywords: “*Humulus lupulus*” AND “health” retrieved 197 total documents, which were subsequently cleaned from the ones that did not have all the searched keywords in the title, abstract, or author keywords. After this manual inspection and filtering process, 135 documents were collected and analysed for the purpose. Successively, the Scopus function “Analyse search results” was used to characterise the obtained bibliographical dataset (year of publication, number of publications, document type, countries/territories of origin, institutions).

The graph about the “Documents by year” (Figure S1a) shows that the research field of “*Humulus lupulus*” AND “health” is a relatively recent topic, with about 20 years of life. It begins to increase on the year 2002 (2 documents), then shows a plateau-like period between 2003 and 2017, ranging from 4 to 7 documents, and a sharp increase in 2018 (8 documents) and 2019 (17 documents). Up until the date of the search (1 September 2022), 13 documents were present in Scopus and this number of publications was probably underestimated because all the calendar years could not be included in the search. The pie graph about “Documents by subject area” (Figure S1b) shows that “Agricultural and Biological Sciences” is the largest topic with 26.1% (68 documents), highlighting the interest of the scientific community on the hop as a crop and its biological characterisation. Most importantly, the graph outlines the major scientific interest in disciplines related to the research on *Humulus lupulus* for health purposes, with an overall 47.2% of the publications (123 total documents) for the topics related to medicine such as: “Medicine” (16.9%–44 documents), “Biochemistry” (15.5%–43 documents), “Pharmacology” (11.1%–29 documents), and “Immunology and Microbiology” (2.7%–7 documents). Interestingly, the top three countries that have shown the greatest scientific interest over the years and contributed to the area of research on *H. lupulus* for health in terms of the number of articles published are: “United States” (32 documents), “Germany” (26 documents), and “Belgium” (14 documents) (Figure S1c), with the first two countries being the largest producers of hops to date (https://www.fao.org/faostat/en/#data/QCL/visualize).

The knowledge structure present in the 135 documents, constituting the bibliographical dataset after the Scopus query, was analysed by constructing and visualizing keyword distance maps based on keyword co-occurrence in VOSviewer. From the 135 documents, the software extracted 2412 keywords, of which 128 met the threshold of minimum occurrences (default setting: 5). The authors decided arbitrarily to exclude from the final calculation the keywords: “article”, “brewing”, “priority journal”, “review”, “united states”, to make the interconnections generated by the software more relevant to the purpose of the analysis. All other VOSviewer parameters were left at default settings.

**Figure 3:**
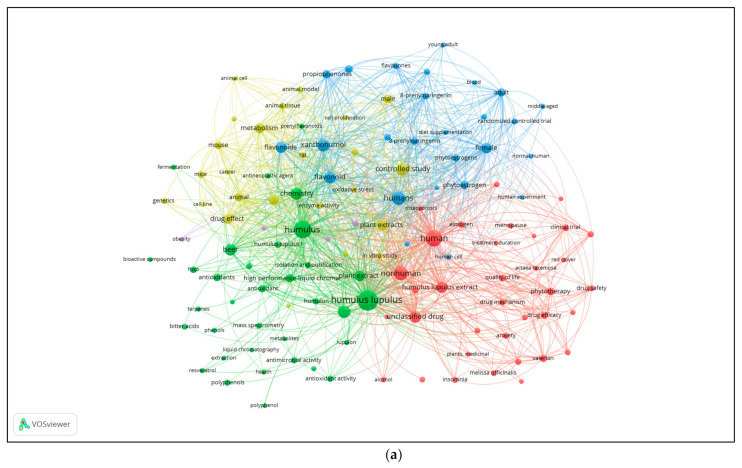

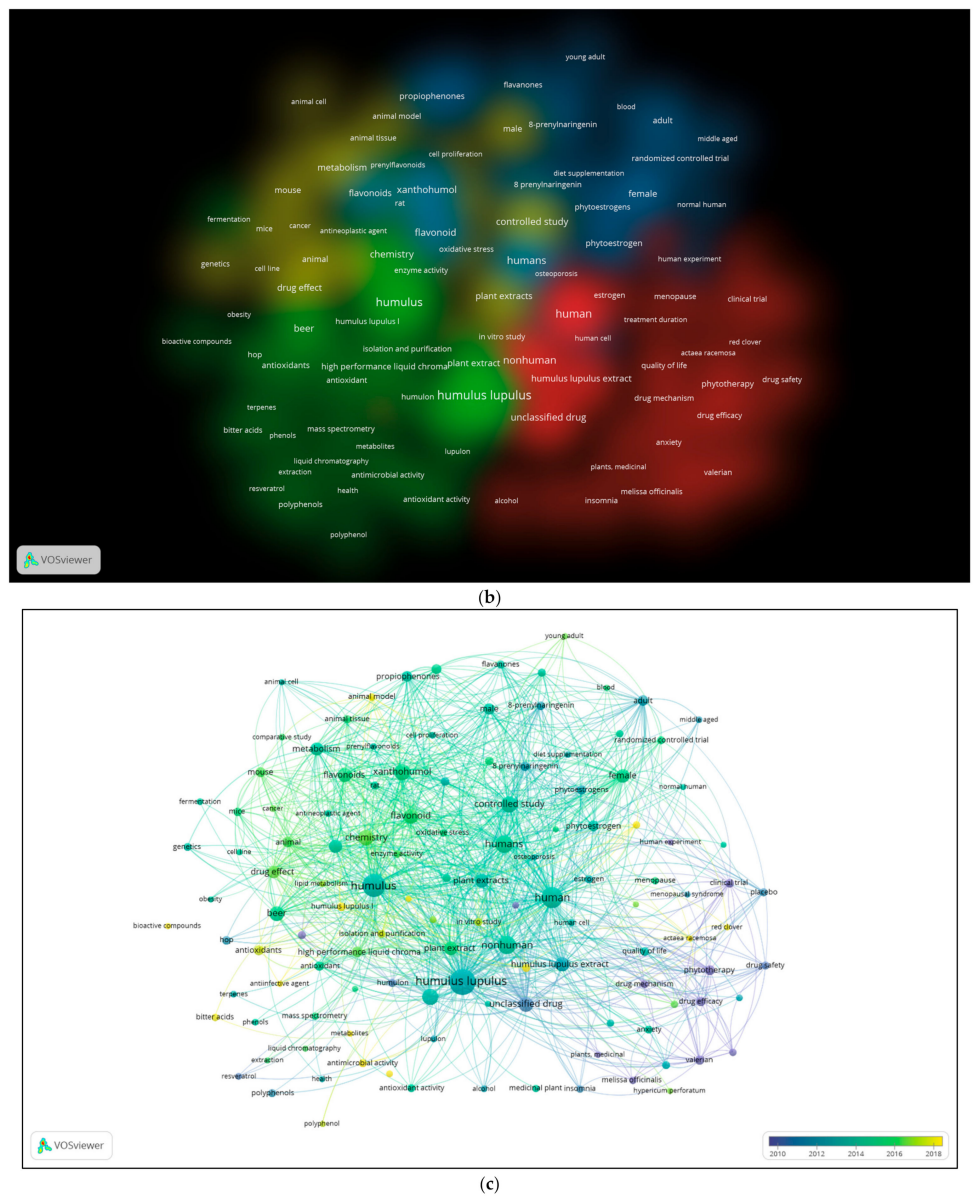
(**a**) “Network Visualization” of the keyword co-occurrence network map generated using VOSviewer. (**b**) “Cluster density” of the map generated in VOSviewer. Each density representing a cluster has a different colour. (**c**) “Overlay visualization” of the keyword co-occurrence network map generated by VOSviewer. The lighter the yellow colour, the more recent the average year of publication of the keywords represented by the nodes. All maps were generated using VOSviewer software from the results of the search performed in Scopus (until 1 September 2022) and subsequent cleaning of the dataset.

The keyword co-occurrence network map or graph shows the connections (links) between the keywords, graphically represented by nodes, occurring in the documents of the dataset obtained from Scopus. Every plotted link between two nodes represents a co-occurrence of two keywords into the same document. The larger the diameter of a node, the higher the absolute number of a keyword occurrence in the overall dataset of analysed documents. VOSviewer identified five clusters (see Table S1 for cluster composition and details), each represented by a different colour in the network map and cluster density map (Figure 3a,b). The network map (Figure 3a) points out the absence of a single central node in the graph, showing several keywords, among the most recurrent ones, represented by several large nodes in almost all clusters, attracting each other in the central area of the map, all highly interconnected with nodes in other clusters. This topology suggests a highly interconnected relationship between the search topics represented by the different clusters. The first cluster (CS_1; red colour) has “human” as the most occurred keyword (n. of occurrences 55), which is characterised by a high number of links with nodes in all the clusters. CS_1 is composed by 35 nodes, which are collectively related to the topic on the use of *Humulus lupus* in phytotherapy alone or in association with other medicinal plants for the treatment of “anxiety”, “insomnia”, “menopause”, “sleep disorder”, “osteoporosis”. In CS_2 (green) the keyword “*Humulus lupulus*” has the largest diameter (87 occurrences); it is very close to the CS_1 and has a high number of links with several nodes in all the clusters. CS_2 is composed by 34 nodes related to the topics that can be summarised as chemical and biological characterization of bioactive compounds from *Humulus lupulus*, as suggested by the keyword “antioxidant”, “antimicrobial activity”, “antineoplastic agent”, “antiinfective agent”. CS_3 (light blue colour), in which the most occurred keyword is “humans” (39 occurrences), is composed of 26 elements. It is not in the centre of its cluster but is very close to CS_1 and again has a high number of links with nodes also in the other clusters. The main topic of CS_3 is composed by research on humans, particularly on the female health and the phytoestrogenic properties of hop extracts. In CS_4 (yellow colour) the most occurred keyword is “controlled study” (32 occurrences) that is in the cloud of CS_3. It is composed by 24 nodes that were extracted by the software to represent in vivo (animal models) and in vitro (cell lines) studies to unravel the “genetics”, “enzyme activity”, “antineoplastic activity”, and “gene expression” changes exerted by hop extracts. Finally, CS_5 (pink colour) is a small cluster consisting of only 4 elements with very few occurrences of each element (5–8) and interspersed at the border with CS_2 and CS_4.

**Figure 4:**
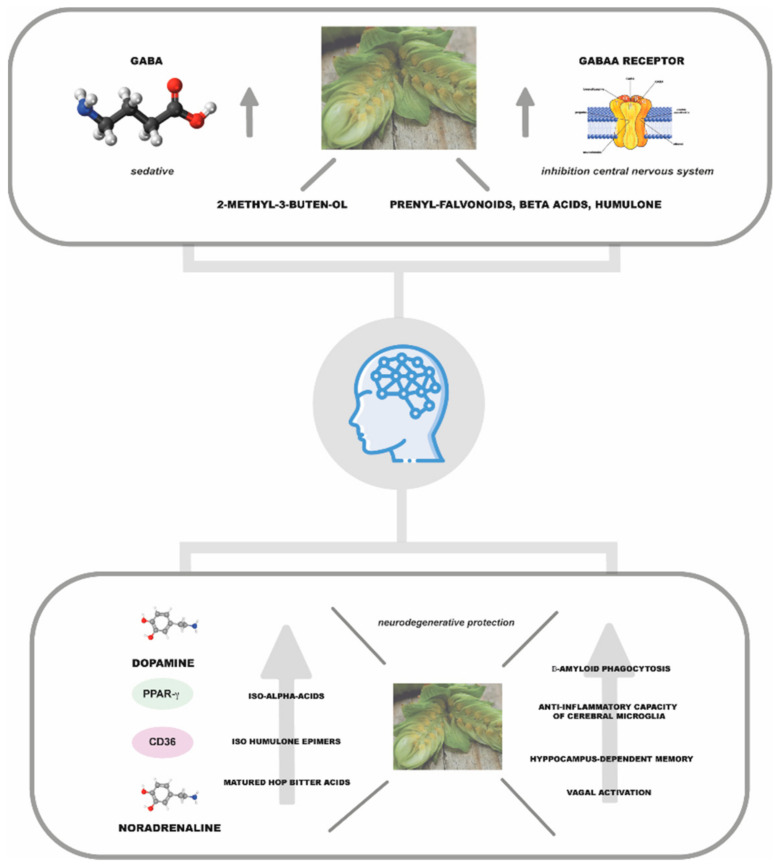
Bioactive compounds and molecular targets involved in the neuroprotective potential of *Humulus lupulus* L. The GABA structure is from https://es.wikipedia.org/wiki/Ácido_γ-aminobutírico (accessed on 21 November 2022); the GABA_A_ receptor is from David M. Lovinger, Public domain, via Wikimedia Commons https://upload.wikimedia.org/wikipedia/commons/3/3a/GABAa_receptor.gif (Last accessed on 21 November 2022). This illustration was designed using images from Flaticon.com.

The network of keyword co-occurrences and the density maps of the clusters (Figure 3a,b) clearly show that all the clusters are not separated by sharp boundaries but instead share a certain degree of overlap, which suggests a strong interdependent relationship between the research themes in this research area. CS_1 shows partial overlap and connection with CS_3, which highlights studies conducted to investigate phytotherapeutic approaches to male and female health using hop extracts alone or in combination with other plant species (“valerian”, “valeriana officinalis”, “melissa officinalis”, “actaea racemose”, “hypericum perforatum”, “red clover”). In addition, CS_3 and _4 show a large overlap (Figure 4), which can be explained by the logical consequential linkage of medical research conducted *in vitro* and *in vivo* and, finally, in controlled studies to be carried out prior to final therapeutic use of any compound. The same observation may also explain the partial overlap observed between CS_2 and _4, as chemical and biological characterisation of any compound usually precedes or is contemporaneous with *in vitro*, on cell lines, and *in vivo*, in animal, studies.

In addition, the “Overlay visualization” generated using VOSviewer (Figure 3c), for which the colour of the nodes highlights the average year of publication (apy) for each keyword analysed, with bright yellow indicating the most recent, points out some very recent research topics in which hop extracts were used in association with “red clover” (apy: 2017), or with “actaea racemose” (apy: 2020) and in which *Humulus lupulus* was studied as an “anti-infective agent” due to its “antimicrobial activity” (both apy: 2018). Lastly, it is worth noting that careful inspection of the maps generated using VOSviewer identified a common error in the spelling of the keyword “8 prenylnaringerin”, which was often used, within the analysed bibliography, instead of the correct one, namely “8-prenylnaringerin” (Figure S2). The generation of two different nodes instead of just one is probably the result of this improper spelling in the name of the molecule in question by some authors, which can, if undetected, generate a bias in the interpretation of the bibliographic network maps, as the software considers the correct and incorrect keywords as if they were two different ones, leading to a change in the topology of the network maps and thus a significant bias in their interpretations.

## 7. Therapeutic Potential of Hops

### 7.1. Sedative and Neuroprotective Activities

Sleep disorders are quite frequent in the population worldwide and may lead to health problems. The use of complementary therapies based on botanical extracts and herbs are evidenced to be a powerful ally, in addition to standard drugs and psychological interventions, for treating insomnia and anxiety [118].

The use of hops in traditional medicine as a mild sedative arose from the experience of drowsiness and chronic forms of fatigue that the pickers and those who worked with or handled hop inflorescences manifested [82]. An ancient folk remedy is the sleeping drink of fresh hop cones, which are also sealed inside a pillow to be held under the head during the night in order to facilitate sleep. The European Medicine Agency classifies hop herbal preparations as “traditional herbal medicinal products” to be used for the relief of mild symptoms of mental stress and to aid sleep [Lupuli flos. European Medicines Agency. Available online: https://www.ema.europa.eu/en/medicines/herbal/lupuli-flos, accessed on 10 August 2022).

In 1980, Hänsel and his collaborators identified the compound to which to ascribe this activity, namely a degradation product formed during the storage process of humulone and lupulone by auto-oxidation: 2-methyl-3-buten-2-ol (Figure 2e) [28]. The molecule, which is a volatile tertiary alcohol, is also found in hop EO [27]. This molecule increases the activity of the neurotransmitter γ-aminobutyric acid (GABA), inhibiting the central nervous system. Franco et al. (2012) demonstrated in a diurnal animal model that the concentration of 2 mg of hop extract effectively decreased nocturnal activity in the circadian rhythm [29]. However, the concentration of 2-methyl-3-buten-2-ol in hops is too low to justify the plant’s sedative activity alone, suggesting synergy with other compounds present in the volatile fraction such as linalool [27]. Moreover, recent literature studies have highlighted the possible role of other phytocompounds in determining the sedative potential of hops (Figure 4) [30]. Among phloroglucinol derivatives, α-acids are considered the main phytochemical class with significant pentobarbital properties, while lupolones mainly express antidepressant and sedative activities [1,6]. It is hypothesised that these sedative effects are mediated by an increase in the function of GABA_A_ receptors, which are responsible for fast-acting inhibitory synaptic transmission in the brain.

Other hop phytoconstituents that possess sedative effects include 8-PN. From a stereochemical point of view, this compound has a single chiral carbon centre in its structure, leading to two enantiomers, with enantiospecific bioactive properties that may significantly influence the pharmacological, toxicological, and pharmacokinetic profile of 8-PN [119]. Bagatin et al. (2014) investigated the effectiveness of a long-term administration (21 d) of a racemic mixture of 8-PN to rats submitted to the elevated T-maze (ETM) model of generalized anxiety and panic disorders. The pharmacological trials demonstrated no effects following an 8-PN acute treatment (10 mg/kg), confirming the existence of a latency period until the onset of the therapeutic action. Moreover, using docking simulations, the authors found that between the two enantiomers, (R)-8-PN had a greater affinity (lower inhibition constant) for all transporters tested (serotonin, norepinephrine, and dopamine ones) than enantiomer (S)-8-PN, indicating that the panicolytic effect observed in the animal model can be due mainly to the R enantiomer of the racemic mixture used [31].

Recently, Benkherouf et al. (2020) proposed that the neuroactivity of hops may involve more than one phytocompound, whose synergistic action leads to enhanced GABA_A_ receptor function. Indeed, the researchers highlighted the role of IX and 6-PN in enhancing the effects exerted by humulone on this receptor [32]. The authors also found that a low dose of humulone (10 mg/kg) increased ethanol- but not pentobarbital-induced sleep duration, also pointing out a non-competitive synergy of humulone with ethanol at GABA_A_ receptors, which could be responsible for further increased alcohol intoxication with high-hopped beers. The synergistic effect in promoting sedative activity, however, is not only observed in the additive action of hop phytoconstituents but has also in the combined action of plant essences such as hops and valerian. In this regard, Morin et al. (2005) reported that a valerian–hop combination and diphenhydramine might be useful adjuncts in the treatment of mild insomnia [120]. In addition, it has been observed that a mixture of valerian and hop (cv. ”Cascade”) demonstrated a higher binding ability on GABA receptor than valerenic acid or/and XN, which are estimated to be active compounds in the extract tested, improving sleep-related behaviours, including sleeping time, by modulating GABAergic/serotonergic signalling [33].

Several studies have investigated the possible role of hop phytoconstituents in the treatment of age-related neurodegenerative diseases (Figure 4). Iso-α-acids have been found to activate peroxisome-proliferator-activated receptor-g (PPAR-g)^2^, a known therapeutic target in Alzheimer’s disease (AD), highlighting a possible role of these compounds on the pathogenesis of AD [34,35]. Ano et al. (2017) demonstrated, in a primary microglia cell culture, that both isohumulone epimers were able to significantly increase β-amyloid phagocytosis by increasing CD36 expression, with trans-isohumulone being more active in this process. In contrast, trans-isoadhumulone and cis-isoadhumulone did not show any significant effect in *in vitro* experiments. Moreover, the authors demonstrated in experiments on an animal model of AD that iso-α-acids administered orally (0, 4, or 20 mg/kg iso-α-acids once a day for 3 days) could penetrate the rat’s brain and that cerebral microglia demonstrated increased anti-inflammatory capacity and β-amyloid phagocytosis [20]. In addition, a single intragastric administration of hop iso-α-acids (0.02–2 mg/Kg), in a rat model, was shown to increase total and extracellular levels of dopamine and its metabolites in the hippocampus, though not in the frontal cortex, in a vagus-nerve-dependent manner, thereby improving hippocampus-dependent memory. The dose equivalent for humans, according to the authors, should be of 0.03–3 mg/Kg, i.e., for a 70 kg person, 2 and 20 mg of active constituents [36].

During storage, hop bitter acids undergo a series of oxidative processes that give rise to oxides generally referred to as matured hop bitter acids, which share with iso-α-acids the β-tricarbonyl moiety (2-acetyl-3-hydroxy-2-cyclopenten-1-one) believed to contribute to vagal activation, also by increasing the levels of noradrenaline [37,38]. These compounds suppressed the activation of microglia and memory impairment observed in AD mouse models, an effect mediated by the noradrenergic system. Albeit with obvious limitations, which will have to be overcome through increased research in this area, these results open up new scenarios towards the use of bioactive hop compounds in the prevention of neurodegenerative diseases.

### 7.2. Antimicrobial and Antiviral Activity

Since ancient Egypt, hops have been used for food preservation and later used for extended storage of beverages, such as beer [82]. The addition of hops reduces the growth of *Lactobacillus*, the main contaminant in beer, which causes losses in ethanol production and the formation of undesirable flavours [121]. Several studies reported an inhibitory activity of bitter acids towards Gram-positive bacteria, such as *Lactobacillus*, *Streptococcus*, *Staphylococcus*, *Micrococcus,* and *Bacillus*, and fungi, such as *Penicillium* and *Aspergillus* species [7,8]. It has been reported that hop bioactive compounds are also active against Gram-negative bacteria such as *Helicobacter pylori* and *Brucella* species [2,8].

Hop bioactive compounds can exhibit either bacteriostatic or bactericidal activity depending on the bacterial growth conditions [9]. In general, among bitter acids, lupulone has greater antimicrobial activity than humulone, which is, in turn, more active than isohumulone. Several molecular mechanisms of action have been proposed to explain these observed effects, the lipophilic region of the bacterial cell membrane being one of the main target sites of hop bitter resins [122,123]. Behr and Vogel (2010) proposed two different mechanisms of hop bacterial inhibition: proton-ionophore-induced and oxidative-stress-induced mechanisms [124]. Michiu et al. (2019) evaluated the inhibitory effects of hop iso-α and -β acids against the bacterium *Pediococcus pentosaceus* at both high (6.0–7.0) and low (4.0–5.0) pH values, testing whether the identified iso-α acid stress altered *S. cerevisiae boulardii* yeast activity and ethanol production [10]. Results pointed out an inhibitory effect of both iso-α and β-acids against *P. pentosaceus* at the pH values tested, opening the possibility of hops being used as a supplement to prevent beverage contamination with spoilage microorganisms.

Several other hop phytoconstituents have shown antimicrobial and antiviral activities. Cermak et al. (2017) tested the antimicrobial activity of purified hop constituents humulone, lupulone, and XN against some anaerobic bacteria of indigenous human flora (*Bacteroides fragilis, Clostridium perfringens, Clostridium difficile*) [9]. Results reported by the authors demonstrated that XN exhibited the highest antimicrobial activity against all three microorganisms, followed by β-acids and α-acids; they also highlighted a different course of inhibitory effects between bitter acids and the prenylflavonoid studied.

Fahle et al. (2022) reviewed the antibacterial activities of humulone, lupulone, and XN, also underlining a synergistic effect when used in combination with antibiotic drugs, not only on Gram+ but also on Gram- bacteria. In addition, the authors pointed out the paucity of *in vivo* studies that could support a real application of these valuable phytochemicals from the laboratory to the clinical setting [11]. Terpenes from hops also demonstrated moderate antimicrobial effects against Gram-negative bacteria (e.g., *Proteus vulgaris*, *Escherichia coli*, *Pseudomonas aeruginosa*, *Salmonella* spp.), and Gram-positive bacteria (e.g., *Enterococcus faecalis* and *Staphylococcus aureus*) [7].

Serkani et al. (2012) evaluated the antimycobacterial effect of ethanolic hop extracts (4 and 8 mg/mL) on rifampin-sensitive and -resistant strains of *Mycobacterium tuberculosis*. The results demonstrated that hop extracts completely inhibited all Mycobacteria strains tested, comparably to rifampin, with minimum inhibitory concentration (MIC) values ranging from 400 to 800 μg/mL, indicating the effectiveness of hop phytocompounds to control tuberculosis *in vitro* [12].

Recently, Blaxland et al. (2022) evaluated fifty aqueous hop extracts from different hop varieties provided both whole and pelleted against *Mycobacterium bovis* BCG, showing that all extracts tested exerted inhibitory activity ranging from 1.2 mm to 15.7 mm depending on the hop cultivar. ”Citra” was the most active, with a MIC and a minimum bactericidal concentration (MBC) of 16% *v*/*v* [13]. An increasing amount of scientific research is focusing on the study of natural compounds as alternative agents to manage the treatment of infections caused by multidrug-resistant bacteria, such as methicillin-resistant *Staphylococcus aureus* and multidrug-resistant *Staphylococcus epidermidis*, which, together with *Cutibacterium acnes*, are the main strains involved in skin diseases. Recently, Di Lodovico et al. (2020) investigated the antimicrobial and antibiofilm properties of an hydroalcoholic hop extract (cv. “Cascade”) against staphylococcal strains and *C. acnes,* including multiresistant isolates. The results highlighted a strong antibacterial action of the extract against the anaerobic *C. acnes* (MIC:1 μg/mL) and a significant reduction in biofilms formed in the presence of subinhibitory concentrations of hop extract [14]. The susceptibility of *P. acnes* and *S. aureus* to a hop CO_2_-extract with 50% humulone and lupulone was tested in vitro (MIC values: 3.1 and 9.4 µg/mL, respectively). The extract also exhibited an additional anti-inflammatory effect by reducing the IL-6 expression (IC_50_: 0.8 µg/mL). The authors also demonstrated the efficacy of a gel formulation with 0.3% of the extract (*w*/*w*) as an antibacterial agent against the two strains tested, significantly superior to the placebo gel [15].

Hop extracts are also reported to inhibit some fungal strains such as *Candida*, *Fusarium*, *Trichophyton,* etc. [2,16]. Yan et al. (2021) reported a moderate antifungal activity of an ethanolic extract of *H. lupulus* against five phytopathogenic fungi (*Rhizoctonia solani*, *Sclerotinia sclerotiorum*, *Botrytis cinerea*, *Fusarium graminearum*, and *Magnaporthe oryzae*; inhibition rate: 37–51% at 500 μg/mL) [17]. Moreover, the authors reported that IX was effective against *S. sclerotiorum*, *F. graminarum*, and *B. cinerea*, for the latter both *in vivo* and *in vitro*. The prenylflavonoid inhibited the spore germination of *B. cinerea* in a dose-dependent manner, causing the destruction of the cell membrane by membrane lipid peroxidation, which caused final damage to the fungal mycelia. Among the hop prenylflavoinoids, 8-PN has been shown to exert an important antifungal activity against *Tricophyton mentagrophytes*, the most common fungal agent in mice, which can also be transmitted to humans, with a MIC equivalent to that of griseofulvin (6.25 mg/L), a natural antibiotic extracted from a species of *Penicillium*, whose antifungal effect is achieved through inhibition of the mitotic process. That being said, the prenylflavonoid demonstrated no inhibitory activity against *Candida* and *Fusarium* strains tested. Similar results were reported by Macchioni et al. (2021), who did not observe any growth inhibition when testing green hop extracts against different *Saccharomyces cerevisiae* (non-pathogenic yeast) strains and against *Candida albicans* (the most common human fungal pathogen), indicating that the effect of these extracts was specific for prokaryotic cells [8].

Jiang et al. (2023) found that hop EO nano emulsion (d < 145 nm) displayed antifungal activity against *F. graminearum* growth and demonstrated mycotoxin-inhibitory activity, suppressing the production of deoxynivalenol. According to the authors, IX exerted its action by altering the total lipid and chitin content in the outer cell membrane and by impairing the permeability of the cytoplasmic membrane [18]. Bocquet et al. (2018) evaluated *in vitro* the antifungal activity of crude extracts of different hop tissues (cones, leaves, rhizomes, and stems; cv. “Nugget”), as well as the EO from the strobile, against *Zymoseptoria tritici,* responsible for Septoria tritici blotch, a devastating foliar disease of wheat. All tested samples demonstrated antifungal activity against *Z. tritici*, relevant only in the case of crude extract and EO, with a decrease of 85% and 100% in colony diameter at 1.25 g/L, respectively. Among purified phytochemicals from crude extract, only co-humulone and DMX demonstrated an inhibitory effect against *Z. tritici* in a dose-dependent manner. In addition, the authors highlighted a synergistic effect against the pathogen by using hop essential oil in combination with the synthetic fungicide bixafen, the antifungal activity of which was improved up to eight times in combination with hop oil [19].

Finally, hop phytochemicals have been demonstrated to also be active against some viruses, such as oral herpes virus infections, influenza, hepatitis C, HIV-1, and SARS-CoV-2 [2,3,4].

### 7.3. Antitumour Activity

Hop bitter acids affect cancer through the induction of controlled cell death in malignant cells [6]. This was first reported by Tobe and coauthors in 1997 when they observed the ability of humulone to induce apoptosis in leukaemia cells [40]. Lupulone, colupulone, and especially hexahydrocolupulone, a semysinthetic derivative of colupulone, inhibit the growth of several human cancer cell lines [41]. Saugspier et al. (2012) analysed the effects of bitter acids on tumorigenicity of hepatocellular carcinoma cells *in vitro*, pointing out that different cellular pathways, such as ERK1/2 phosphorylation, regulation of AP-1 activity, and nuclear factor NF-κB, are the main targets of the antiproliferative action of bitter acids, with β-acids more active than α-acids [125]. Furthermore, Lin et al. (2019) examined the antitumour potential of tetrahydro-, hexahydro-iso-α-acids, and rho-iso-acids (prepared from a modified hop extract), highlighting the ability of the latter to interfere with the prostaglandin E2 metabolic pathway by inhibiting its biosynthesis [39].

As already pointed out in the previous section, bitter acid oxidation compounds, present in greater quantities in hop pellets as they are more exposed to oxygen during the production process, may also potentially play an important role in cancer treatment, as adjuvants in chemotherapeutic protocols. In this regard, Salviati et al. (2019) demonstrated that a subfraction of a hexane extract of hop pellets, consisting mainly of humulinones and cohulupone derivatives, can act as a stimulator of natural killer (NK) cells, inducing, at a dose of 0.1 mg/mL, the selective activation of the NKp44 receptor, while simultaneously enhancing the cytolytic activity of NK cells against the leukemic K562 cell line. The authors also pointed out that the observed effect was dose-dependent, as higher-dose treatment (1 mg/mL) significantly reduced NKp44 expression [42].

Over the years, attention has been focused on the antitumour and chemo-preventive potential of XN as a multi-target compound able to modulate in a different way several signalling pathways and compounds involved in tumorigenesis [59,126]. Its action is dose-dependent, inducing a cytoprotective mechanism at low concentrations (0.01 µM), while leading to apoptosis and cellular cytotoxicity at higher concentrations (5 µM) [43].

Hsieh et al. (2022) investigated the apoptotic effect and anticancer properties of XN in human nasopharyngeal carcinoma cell (NPC) lines. Results indicated that XN effectively induced the upregulation of the c-Jun N-terminal kinase in the mitogen-activated protein kinase, promoting the apoptosis of NPC [44]. Recently, several authors have also suggested that the anticancer properties of XN may be due to its prooxidant activity, as it is able to induce ROS generation through NADPH oxidase in a dose-dependent manner [126,127,128]. Blanquer-Rossellò et al. (2013) found that XN increased ROS levels in breast cancer cells by improving mitochondrial function at high concentrations [43]. Moreover, Stevens (2020) found that XN can inhibit cytochrome P450 enzymes, which metabolically activate procarcinogens and induce the carcinogen-detoxifying quinone reductase and pro-angiogenic pathways [45]. Cancer cells are characterized by a higher level of ROS than healthy ones and, consequently, their ROS threshold for apoptotic induction is higher; in this regard, XN may be a promising chemotherapeutic agent, especially if used in combination therapy, since malignant cells cannot develop resistance against the mitochondrial uncoupling effects of prenylated flavonoids [45].

From a critical analysis of the literature, it appears that the mechanisms underlying the anticarcinogenic action of XN are related to the inhibition of two signalling pathways implicated in the onset of malignancy and the metastatic process: Akt (or PI3K-Akt) signalling pathway that promotes a cell’s survival and growth in response to extracellular signals, and NF-κB (nuclear factor kappa light chain enhancer of activated B cells), a family of highly conserved transcription factors that regulate many important cellular behaviours, in particular inflammatory responses, cellular growth, and apoptosis [46]. In addition, the antimetastatic effect of XN also appears to be due to the inhibition of the MAPK/ERK pathway (also known as the Ras-Raf-MEK-ERK pathway) [47]. The anticancer action of XN has also been reported for several tumour types such as gastric and colorectal cancer [129]. In this regard, Turdo et al. (2021) highlighted the synergistic effect of XN in enhancing the efficacy of nobiletin, a polymethoxyflavone derived from *Citrus sinensis*, in suppressing colorectal cancer stem cells, underlining the adjuvant potential of this mixture in cancer therapies. The authors found that this mixture of bioactive compounds was able to suppress the migration of cancer stem cells, reducing the expression of CD44v6 and inducing apoptosis and cycle arrest in the G2/M phase [130].

Among hop prenylflavonoids, not only XN has attracted the attention of scientists for anticancer potential. Wang et al. (2016) tested an enriched hop extract containing 1.2% 6-PN, 0.33% 8-PN, 0.99% IX, and 32% XH and its pure bioactive compounds for their effects on oestrogen metabolism in breast cancer cells (MCF-10A and MCF-7). Results from this study provided *in vitro* evidence that hop and its compound 6-PN preferentially induced the nontoxic oestrogen 2-hydroxylation pathway in the two different breast cancer cell lines tested, indicating a potentially protective role of hop in reducing the risk of breast cancer through oestrogen metabolism modulation. Interestingly, the authors found that only 8-PN demonstrated slight up-regulation of metabolism in MCF-7 cells, whereas IX and XN did not have significant effects in either cell line [48]. Krajnovic et al. (2022) evaluated the anticancer potential of XN (5% *w*/*w*) and IX (3.5% *w*/*w*) loaded into mesoporous silica nanoparticles (as delivery/targeting system; pore diameter: 5.44 nm) against malignant mouse melanoma B16F10 cells, underlining that the main mechanism against tumour cells includes inhibition of proliferation and autophagic cell death [49]. Hajirahimkhan et al. (2022), in their preprint, demonstrated that 8-PN suppresses aromatase expression in postmenopausal women’s breast tissue, thus standing for a key role in breast cancer prevention for high-risk postmenopausal women [50].

Recently, Ramazzina et al. (2022) also investigated the potential antitumour effects of hop bioactive compounds on cell viability, intracellular ROS production, and phase II enzyme activation, comparing the results with those obtained by treating Caco2 cells with Polyphenon E, a standardized green tea extract approved by the Food and Drug Administration (FDA). The authors highlighted the role of the extraction process and the chosen solvent on the biological properties of hop extracts towards target cells, explaining the observed in terms of the inhibitor–promoter pair using chemometric models. The results demonstrated the crucial role of molecules such as ferulic acid (promoting)–adlupolone (inhibitor), and coumaric acid (promoting) + protocatechuic acid (inhibitor), respectively [128].

In addition, Rutnik et al. (2021) examined the antitumour potential of hop EO, highlighting the most intense activity of b-caryophyllene and b-caryophyllene oxide [27]. EO and hydrolate from hop cones (cv. “Chinook”) were also tested *in vitro* to investigate their apoptotic potential against several cancer cell lines (human acute promyelocytic leukaemia cells, human neuroblastoma cells, human metastatic mammary adenocarcinoma cells, human mammary adenocarcinoma cells, and a normal mammary epithelial cell). The results demonstrated that the hydrolate was less active than the corresponding EO, which demonstrated greater selectivity against the HL60 leukemic cell line than the other cancer cell lines tested, while also showing cytotoxicity on the normal cell line. In addition, the results demonstrated the lower cytotoxic activity of hop extracts compared with hemp extracts on virtually all cell lines tested [131].

### 7.4. Antioxidant Activity

Hops contain several biomolecules with high antioxidant potential such as flavonols, found in the strobili mainly in glycosidic form, such as rutin (quercetin-3-rutinoside) and astragalin (kaempferol-3-glucoside) [61].

In 1995, Tagashira et al. demonstrated that hop bitter acids possess a high antioxidant potential and also a lipid peroxidation inhibitory activity [132]. Recently, Wang et al. (2022) found that the antioxidant activity of β-acid extracts with different contents of colupulone (30%, 50%, 70%, 90%, and 100%) differed in a dose-dependent manner [74]. In addition, Yang et al. (2020) demonstrated that a mixture of colupulone and n-lupulone + ad-lupulone (1:4) possessed greater antioxidant activity than colupolone alone [24]. In general, α-acids exhibit higher scavenging potential than the corresponding iso-α-acids towards both OH radicals and lipid peroxidation [25]. Wang et al. (2014) evaluated the antioxidant activity of a high purity hop polyphenol extract (total phenolic content: 887 mg/g) both *in vitro* and *in vivo*, comparing their results with the effects produced by green tea polyphenols. The authors found that the scavenging effects of hop polyphenols on DPPH, •OH, and O2^•−^ radicals were superior to those of tea ones and that hop polyphenols were able to counteract oxidative DNA damage in a dose–response manner over the range of concentrations tested (0.1–0.3 mg/mL). *In vivo* trials on mice pointed out that a hop polyphenol dose of 400 mg/kg body weight demonstrated the most beneficial effects in terms of protection from bromobenzene-induced lipid peroxidation in mouse liver, highlighting a protective effect of these polyphenols on antioxidant enzymes (i.e., superoxide dismutase and glutathione peroxidase) from damage by reactive oxygen species *in vivo* [133].

Recently, Santarelli et al. (2022) demonstrated that ultrasound-assisted extractions of hop cones (cv. “Cascade”) provided samples characterized by the highest antiradical capacity, assessed using both ABTS and DPPH assays [109]. The authors demonstrated that not only the type of extraction technique applied, but also the process parameters (i.e., temperature, extraction time, ultrasound power) statistically influenced the antiradical capacity of the extracts produced. Similarly, Macchioni et al. (2021) found that tailor-made natural deep eutectic solvents profoundly affected the antiradical capacity of hop extracts. In the study, the authors found that the eutectic solvent mixture lactic acid: glycine (molar ratio 3:1) was able to produce hop extracts with the highest values of antiradical capacity compared to all the samples tested [8].

Interestingly, other plant tissues besides cones also help in defining the antioxidant potential of hops. In this regard, Maietti et al. (2017) evaluated the antioxidant potential of wild hop shoot samples from different locations. Samples analysed demonstrated an antiradical capacity, expressed as mg of Trolox equivalents per g of shoots (fresh weight), ranging from 0.68 to 1.07 mg/g, depending on the location considered [112].

Bitter acids and polyphenols are not the only ones responsible for the antioxidant potential of hop cones. Maliar et al. (2016) analysed the antioxidant capacity of methanol extracts of eight Czech hop cultivars and found that DPPH• did not correlate with any compounds in the cone extracts, while ABTS radical scavenging correlated highly with α-bitter acid content, DMX, and hop EO, but not with total phenolics and total flavonoid content. The latter families of compounds, however, were well correlated with the results from the FRAP assay. Among the cultivars analysed, the best scavenging ability was exerted by Saaz Late, while Agnus was the best in reducing ions of the transition metals tested [117]. Regarding the antioxidant potential of prenylflavonoids, in their very interesting review of the biological activity on 8-PN, Pohjanvirta and Nasri (2022) pointed out some discordant literature data. The main critical issues seem to be related to the type of analytical assay used, particularly the nature of the radical employed. The authors also pointed out a dose-dependent antioxidant activity of 8-PN, on average lower than that of XN [134]. Finally, Kontek et al. (2021), in their recent study, fractionated the powder of the freeze-dried hop cone (cv. “Marynka”), obtaining a fraction (A) rich in bitter acids, both humulones and lupulones, and a second fraction (B) dominated by XN and α-acids, characterised by a different antiradical potential, which was, however, rather moderate for both fractions. Fraction A demonstrated an overall higher antioxidant potential than fraction B; however, both fractions were able to attenuate oxidative stress by 65–95% in lipid peroxidation and protein carbonylation tests [26].

### 7.5. Estrogenic Activity

Research on the estrogenic activity of hops arose from the observation on the occurrence of menstrual disorders commonly contracted by hop cone pickers, to the extent that they had the onset of their cycle two days after the start of inflorescence harvesting, regardless of the time of the cycle they were in [135]. The World Health Organisation reports that the component showing estrogenic activity is 8-PN, the most potent phytoestrogen known to date [136]. It has been reported that the estrogenic activity of hops corresponds to the presence of the equivalent of 20–300 g 17-β-estradiol/g [137]. Particular attention has also been paid to IX, which is a methylated derivative of 8-PN. This molecule has no estrogenic activity; however, *in vivo,* it is demethylated by bacteria in the intestinal flora, leading to 8-PN, which implies that hop products may be sources of more 8-PN than that present in the cone itself, thus increasing its estrogenic potential [134].

The degree of estrogenic activity of a molecule is generally determined by the affinity of the compound for the estrogenic receptors. Most phytoestrogens show a preference for ERβ; in contrast, 8-PN binds predominantly to ERα and is classified as a selective natural estrogenic receptor modulator [53,54]. Moreover, it has been reported that both 8-PN enantiomers displayed high affinity and selectivity for ERα, but S-8-PN exhibited an overall higher affinity for both receptors than R-8-PN, using recombinant human oestrogen receptor (ER)-α and ER-β from cytosolic SF9-cell extracts [55]. Other structurally related hop flavonoids, such as 6-PN, have little estrogenic activity [52]. DMX, on the other hand, is considered a pro-estrogenic because it can isomerise, giving 8-PN, and, unlike it, does not activate hormone receptors [138]. Due to their like-oestrogen activity, hop prenylflavonoids may be suitable to compensate for the reduced levels of 17-β-estradiol during menopause [139]. However, Zanardi et al. (2022) suggested caution in the use of hop preparations as an alternative to hormone replacement therapy to alleviate postmenopausal symptoms, due to the role of oestrogen compounds in the development of endometrial cancer. In this regard, the authors demonstrated, through *in vitro* experiments on Ishikawa cell lines, that 6-PN and hop extract activate the ERa receptor and aryl hydrocarbon (AHR) signalling pathways, with 6-PN being able to increase tumour suppressor gene expression and the expression of genes involved in oestrogen metabolism, by upregulating the expression of cytochrome P450 1A1 (CYP1A1), which is involved in the oestrogen detoxification mechanism, to a greater extent. Of the samples tested, neither hop extract nor 8-PN were able to act on the detoxification pathway, but only on the genotoxic pathway, highlighting the key role of 6-PN as a potential modulator of oestrogen metabolism by virtue of its ERa and AHR agonistic activity [56].

### 7.6. Other Bioactivities

Thanks to its plethora of bioactive constituents, hops have been evaluated for their bioactive potential in the treatment of metabolic disorders [140]. It has been shown through experiments on insulin-deficient diabetic mice that 8-PN can act as an ERa agonist in the regulation of glucose homeostasis and also protect, with an effect comparable to that of naringenin, the pancreas from cell apoptosis and inflammatory responses [141]. Furthermore, XN and its hydrogenated derivatives can be considered a drug candidate for the treatment of metabolic syndrome by having a good anti-obesity activity, inhibiting differentiation of preadipocytes and inducing mature adipocyte apoptosis, thereby decreasing the risk of hypercholesterolemia and dyslipidaemia [142]. Improvements in metabolic syndrome by XN derivatives are linked to altered gut microbiota and bile acid metabolism [143]. Recently, Ponticelli et al. (2021) reviewed the potential of iso-α-acids as adjuvant in metabolic syndrome treatment, acting as agonists on peroxisome proliferator-activated receptors, which play a regulatory role in energy homeostasis and metabolic function [144]. Hop bitter acids stimulate salivation and secretion of gastric juice, as well as secretion of mucopolysaccharide-rich mucus, thereby facilitating digestion and absorption of food, consequently increasing appetite [106].

Treatment with crude hop extract, enriched in bitter acids, may modulate early satiety, which is associated with impaired gastric accommodation and gastric emptying, as these substances are potent ligands for human bitter taste receptors (T2R), with activation thresholds as low as 3 nM [145].

In addition, *in vitro* and *in vivo* studies highlight important biological properties of hop bitter acids and derivatives, including inhibition of bone resorption and anti-inflammatory (COX-2 inhibitory activity) activities [1,140].

Regarding prenylflavonoids, XN has been evidenced to be a drug candidate for the treatment of diabetic skin ulcers by increasing Nrf2 activation. The authors observed that, in a diabetic animal model, 2.5 mM XN was able to increase collagen deposition, promoting the activation of Nrf2 both by AMPKa activation and Keap1 cystein modification [146]. Finally, Frackowiak et al. (2010) found that hop extracts rich in sugar alcohols (mainly *myo*-inositol) and organic acids (i.e., glucuronic and malic acids) were effective in dissolving kidney stones, while also being non-mutagenic and non-cytotoxic [147].

## 8. Pharmacokinetics of Bioactive Hop Compounds

One of the key aspects for the real application of hop-related active ingredients in the treatment of different diseases is related to the availability of information on the pharmacokinetics of its phytoconstituents (i.e., absorption, distribution, metabolism, and excretion), which unfortunately is still rather latent to date.

Regarding bitter acids, their bioavailability, as well as that of their derivatives (iso-α-acids, dihydro-iso-α-acids, tetrahydro-iso-α-acids), has been assessed both *in vitro*, investigating the epithelial transport of these acids across Caco-2 monolayers, and *in vivo,* after beer consumption [148,149].

Catoor et al. (2010) pointed out that, at the doses tested (50 mM, corresponding approximately to the average bitter acid content found in beers obtained by dry hopping), no phase II metabolites were found for α-acids, while a recovery of β absorption of the lipophilic β-acids is limited by substantial glucuronidation and/or sulfation by the enterocytes. In addition, the authors highlighted the passive diffusion of α-acids as the key feature of their permeation through intestinal cells, not only in Caco-2 monolayers but also *in vivo*. The results obtained in this study show that the different lipophilicity of humulones compared with lupolones, due to the different molecular nature of the side chain at C-1 (isopentanoyl in n- and ad-, isobutyroyl in co-analogues), is reflected in substantial differences in diffusion and transport rates [148]. Hop bitter acid phase I metabolism catalysed by cytochrome P450 enzymes was also investigated by incubation of α-, β-, and iso-α-acids (20 μM) with human and liver microsomes isolated from rabbits. The results indicated that, in the experimental conditions adopted (incubation time: 120 min), β-acids were totally metabolized, followed by α-acids (77%), and iso-α-acids (48%). Interestingly, the results obtained indicated that bitter acids and their derivatives exhibited a susceptibility to microsomal oxidative degradation, giving rise to biotransformation products very similar to those found in aged beer (i.e., humulinones) [150].

As for prenylflavonoids, IX, which is more abundant in hops than 8-PN, is converted (up to 4 mg/L) to the latter through the activities of intestinal *Eubacterium limosum*, with an efficiency of up to 36%. In addition, hepatic cytochrome P450 enzymes convert IX into 8-PN [151]. However, it was also observed that selected strains of *E. limosum* can convert up to 90% of 8-PN into IX [152]. Data in the literature highlight a low oral bioavailability and large interindividual variation, in humans, for 6- and 8-PN from hop extracts [153]. However, Calvo-Castro et al. (2018) in a double-blind, placebo-controlled crossover study of 16 healthy volunteers (eight women and eight men), who were given a single oral dose of 500 mg of 6-PN or 8-PN, demonstrated a higher bioavailability of 8-PN compared to 6-PN, which, however, showed similar efficacy to 8-PN in improving peripheral blood mononuclear cell viability [154].

Regarding XN, its bioavailability after oral administration is rather low. Pang et al. (2007) reported that the poor oral bioavailability of this compound observed *in vivo* is mainly due to its specific binding to cytosolic proteins of intestinal epithelial cells and rapid metabolization by microorganisms in the colon [155,156]. Several studies have shown that, upon absorption, hop prenylflavonoids are rapidly metabolised into their corresponding glucuronide and sulphate derivatives, preventing the free forms from reaching the cells [156,157]. XN appears to show a biphasic absorption trend, with peak contraction observed between 1 and 7 h after ingestion, suggesting enterohepatic recirculation [156]. In their study on the tissue distribution and pharmacokinetics of 8-PN versus naringenin in mouse models, Tanaka et al. (2021) found the highest accumulation of 8-PN in the kidney and a significant accumulation in skeletal muscle. The authors also observed that 4 h after the ingestion of an average daily dose of 4.4 g/day/mouse of 8-PN, its plasma concentration increased and remained high up to 8 h after ingestion, being detectable even 24 h after and undetectable only after 48 h [158].

## 9. Human Studies and Clinical Trial Evidence of Hops

One of the most critical issues in the field of botanicals concerns the centrality of clinical research for the development of herbal medicines, which is often lacking and unable to unequivocally verify the efficacy of the natural remedy against a given disease, either in the preventive phase or as an adjuvant in therapeutic treatment.

Regarding hops, Lucas et al. (2021) selected some clinical studies on hops for the treatment of various diseases [159]. Recently, Hurth et al. (2022) developed lipid-rich creams (oil-in-water (O/W) and a water-in-oil (W/O) emulsions) containing a bitter-acids-enriched CO_2_ hop extract (1%, 10 mg/mL), testing them in a randomized placebo-controlled double-blind UVB erythema study with 40 healthy volunteers, using hydrocortisone acetate (1%) as the positive control. The results demonstrated that only the O/W cream formulations exhibited an *in vivo* anti-inflammatory effect in the UVB erythema tests, probably due to the presence of penetration enhancers (e.g., propanediol) and the specific chemical profile of the extract in terms of solubility and lipophilicity of the bioactive components present in the galenic formulation tested [160].

Dabrowski et al. (2022) conducted a clinical trial on 50 adult patients affected by acute respiratory failure caused by the COVID-19 infection, treating a group with an extract from *H. lupulus* rich in XN as adjuvant therapy (three times a day every 8 h at a dose of 1.5 mg/kg body weight, for 7 days) [161]. The authors observed that the XN-enriched extract significantly reduced the severity of the inflammatory response, decreasing plasma serum interleukin 6 concentration and neutrophil-to-lymphocyte ratio; it also improved outcomes and reduced mortality rate. In addition, XN at a daily dose of 4.5 mg/kg body weight improved the oxygenation index and reduced the duration of mechanical ventilation. If confirmed, these data would open new possibilities of use for this medicinal plant.

Walker and co-authors (2019) evaluated, on 30 men, in a placebo-controlled trial, the effect of co-therapy with anorexigenic agents (bitter hops-based appetite suppressant (Amarasate^®^), high dose: 250 mg; low dose: 100 mg) on the subjective ratings of appetite during the 16–24 h period of a day-long water-only intermittent fast. The results demonstrated that bitter acids may regulate appetite independently of meal timing [162]. In addition, a small, placebo-controlled, clinical study was conducted by Ferk and coauthors [163], highlighting that a daily intake of XN (12.0 mg/P/day over a period of 14 days) can reduce oxidative-stress-induced DNA damage. Interestingly, the authors found no alterations of progesterone and 17ß-estradiol concentrations at the end of the trial. A randomized (1:1), placebo-controlled trial was conducted to evaluate the effect of a hop dry extract (Melcalin^®^ 400 mg once daily) on depression, anxiety and stress levels in young adults [164], showing a significant reduction in the levels of these self-reported symptoms, with no significant changes in cortisol concentrations over time in either group. Human studies on a phytochemical-based anti-inflammatory product, consisting of a combination of hop iso-alpha rho acid (200–225 mg per tablet), rosemary, and oleanolic acid, have shown a significant decrease in pain in subjects suffering from chronic inflammation (e.g., osteoarthritis, rheumatoid arthritis, or other autoimmune conditions), probably due to its effect on inhibiting inflammatory signal transduction, leading to the reduction of inflammatory cytokines and prostaglandins such as PGE2, while no gastrointestinal or cardiovascular side effects were reported as in the case of traditional cyclooxygenase (COX)-2 inhibitor administration [165].

Recently, Kenda et al. (2021) reviewed studies in the literature about the *in vivo* estrogenic activity of hop and its prenylflavonoids; they pointed out discordant results but, above all, emphasized the lack of quality of the results produced, which did not allow firm conclusions to be drawn on the effect of hops on menopausal symptoms [166]. In this regard, van Breemen et al. (2021) conducted a phase I study, approved by the Institutional Review Board of the University of Illinois and reviewed by the National Centre for Complementary and Integrative Health, on 16 women selected from healthy peri-menopausal and post-menopausal states, aged from 40 to 79 years old. The results demonstrated that the hop-based dietary supplements for menopausal women (administered orally in the form of capsules (59.5 mg of extract per capsule), each containing 0.25 mg of 8-PN, 1.30 mg of 6-PN, 0.80 mg of IX and 21.3 mg of XN) did not produce any harmful drug interactions, with regard to the enzymes examined during the investigation and limited to the experiment setup [167].

## 10. Conclusions

The critical review of the literature presented here, which focuses on the most recent studies, highlights the enormous therapeutic potential of hop phytochemicals, but also the important critical issues for which an adequate scientific response is urgently needed. Although there is a great deal of research on their biological activities, highlighting their potential both as individual compounds and in synergy with each other, to date clinical and pharmacokinetic studies on hop phytocompounds are still rather sparse and not very robust. The main criticisms inherent in the clinical studies concern the small number of volunteers tested, the strong gender imbalance, and the use of non-standardised hop extracts. It is important to note that several parameters such as genotype, soil, climatic conditions, and post-harvest treatments to which the hops are subjected play a key role in defining the biochemical profile of the phyto-complex. These aspects play an essential role, since the estrogenic potential of some hop compounds may have adverse effects on human health. Therefore, studies on the administration of these extracts to women as adjuvants for the treatment/prevention of various diseases should also consider these effects. The use of extracts, whose entire composition is not defined, may be subject to serious misinterpretation. *In vivo* pharmacokinetic studies, on the other hand, point out as a major criticism the prevalent use of animal models, generally mice, which do not allow for the essential role played by the human microbiota in the metabolism of these xenobiotics. The ability of scientists to address these challenges will determine the real applicability of these valuable molecules in the future.

## Figures and Tables

**Table 1:** Therapeutic potential of hops and related pure compounds.

Therapeutic Properties	Biological Target	Bioactive/Dosage Form	References
Antiviral	DNA; RNA viruses	crude extract, humulone, XN ^1^	[2,3,4,5]
Antibacterial	Gram+; Gram−; anaerobic bacteria	bitter acids, iso-α-acids, XN ^1^, hydroalcoholic extract	[2,6,7,8,9,10,11,12,13,14,15]
Antifungal	*Candida, Fusarium; Trichophyton; Rhizoctonia solani; Sclerotinia sclerotiorum; Botrytis cinerea; Magnaporthe oryzae*	ethanolic extract, IX ^2^, 8-PN ^3^, essential oil nano emulsion, crude extract, essential oil	[2,8,16,17,18,19]
Anti-inflammatory	Toll-like receptor 4; NF-kB signaling IL-1b; IL-6; IL-8; TNF expression	humulone, lupulone, XN ^1^, tetrahydro-iso-α-acids, bitter acids	[15,20,21,22,23]
Antioxidant	ROS; RNS; lipid peroxidation	crude extract, bitter acids	[24,25,26]
Sedative and neuroprotective	GABA_A_ receptors; b-amyloid phagocytosis; dopamine levels; vagal activation; noradrenaline levels	2-methyl-3-buten-2-ol, bitter acids, 8-PN ^3^, 6-PN ^4^, IX ^2^, iso-α-acids, matured hop bitter acids	[1,6,20,27,28,29,30,31,32,33,34,35,36,37,38]
Anticancerogenic	ERK1/2, AP-1, NF-kB, prostaglandin E2, NK cells, NKp44 receptor, JNK, ROS levels, cytochrome P450 enzymes, Akt, MAPK/ERK, CD44v6, aromatase expression, Keap1-Nrf2	Humulone, hexahydrocolupolone, bitter acids, rho-iso-acids, humulinones, XN, 6-PN, IX	[6,39,40,41,42,43,44,45,46,47,48,49,50,51]
Estrogenic	ERa, AHR receptor, CYP1A1	8-PN, 6-PN, crude extract	[52,53,54,55,56]

^1^ XN: xanthohumol; ^2^ IX: isoxanthohumol; ^3^ 8-PN: 8-prenyl naringenin, ^4^ 6-PN: 6-prenylnaringenin.

**Table 2:** Average chemical composition of dried hop cones.

Compound	Hop Cone Content (% *w*/*w* on Dry Basis)
Water	10.0
Resins	15.0–30.0
Essential oils	0.5–3.0
Polyphenols	3.0–14.0
Sugars (monosaccharides)	2.0
Pectins	2.0
Amino acids	0.1
Proteins (N × 6.25)	15.0
Lipids and wax	3.0
Ash	8.0
Cellulose and lignin	40.0

## Data Availability

Data is contained within the article or Supplementary Materials.

## Supplementary Materials

The following supporting information can be downloaded at: https://www.mdpi.com/article/10.3390/plants11243434/s1, Figure S1: (a) trends in the quantitative distribution of published documents per year (“Documents by year”); (b) pie chart of the percentage of documents classified by subject area. (“Documents by subject area”); (c) number of published documents by country (“Documents by country or territory”). Statistics on the quantitative distribution of papers published in Scopus after querying: “*Humulus lupulus*” AND “health”, up to 1 September 2022. All the charts were generated with the “Analyse search results” function; Figure S2: Detail of keyword co-occurrence network map generated by VOSviewer software to spot the keyword mistake of “8-prenylnaringerin” in the cluster 3. Table S1. Cluster composition of keyword co-occurrence network analysis from the cleaned dataset after Scopus search: “*Humulus lupulus*” AND “health”. Order of the keywords listed in each cluster: number of occurrences.
