# Supplementary material for: An Updated Review of the Genus Humulus: A Valuable Source of Bioactive Compounds for Health and Disease Prevention
Source: Plants (Basel). 2022 Dec 8;11(24):3434. doi: 10.3390/plants11243434 (PMC9782902; doi:10.3390/plants11243434)
Supplement: Supplementary file 1 [file plants-11-03434-s001.zip › SUPPLEMENTARY_MATERIALS_CARBONE_revised/TABLE_S1.pdf]

Table S1. Cluster composition of keyword co-occurrence network analysis from the cleaned dataset after Scopus search: "*Humulus lupulus*" AND "health". Order of the keywords listed in each cluster: number of occurrences.

**Cluster Number (colour)**	**Number of keywords**	**Selected keywords (In order of number of occurrences)**
1 (red)	35	human; nonhuman; unclassified drug; humulus lupulus extract; phytotherapy; drug efficacy; valerian; clinical trial; quality of life; anxiety; drug mechanism; drug safety; estrogen; valeriana officinalis; phytochemical; placebo; dietary supplement; herbaceous agent; herbal medicine; insomnia; medicinal plant; melissa officinalis; menopause; sleep disorder; alcohol; liver toxicity; actaea racemose; estrogen activity; hypericum perforatum; menopausal syndrome; osteoporosis; plants, medicinal; red clover; treatment duration; women's health
2 (green)	34	humulus lupulus; humulus; hops; chemistry; beer; plant extract; high performance liquid chromatography; antioxidants; antioxidant; hop; humulus lupulus l; humulon; isolation and purification; antimicrobial activity; antioxidant activity; chromatography, high pressure liquid; polyphenols; bitter acids; mass spectrometry; antineoplastic agent; essential oils; fermentation; lupulon; phenols; prenylflavonoids; resveratrol; terpenes; antiinfective agent; bioactive compounds; extraction; health; liquid chromatography; metabolites; polyphenol
3 (light blue)	26	Humans; xanthohumol; flavonoid; flavonoids; female; adult; phytoestrogen; propiophenones; propiophenone derivative; 8 prenylnaringenin; phytoestrogens; 8-prenylnaringenin; isoxanthohumol; flavanones; flavanone derivative; randomized controlled trial; dietary supplements; bioavailability; diet supplementation; human cell; middle aged; blood; cytotoxicity; human experiment; normal human; young adult
4 (yellow)	24	controlled study; plant extracts; metabolism; animals; drug effect; male; animal; mouse; animal experiment; mice; animal tissue; genetics; in vitro study; rat; animal model; enzyme activity; oxidative stress; antineoplastic activity; cancer; cell proliferation; comparative study; animal cell; cell line; gene expression
5 (pink)	4	chemical structure; lipid metabolism; obesity; phytochemicals
