# Supplementary figures and images for: An Updated Review of the Genus Humulus: A Valuable Source of Bioactive Compounds for Health and Disease Prevention
Source: Plants (Basel). 2022 Dec 8;11(24):3434. doi: 10.3390/plants11243434 (PMC9782902; doi:10.3390/plants11243434)

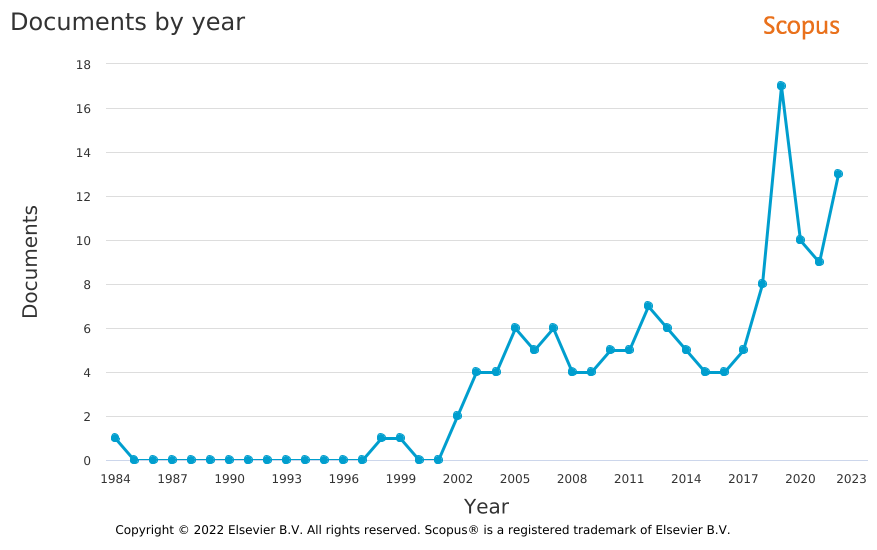

Supplement: Supplementary file 1 [file plants-11-03434-s001.zip › SUPPLEMENTARY_MATERIALS_CARBONE_revised/FIGURE_S1a.png]

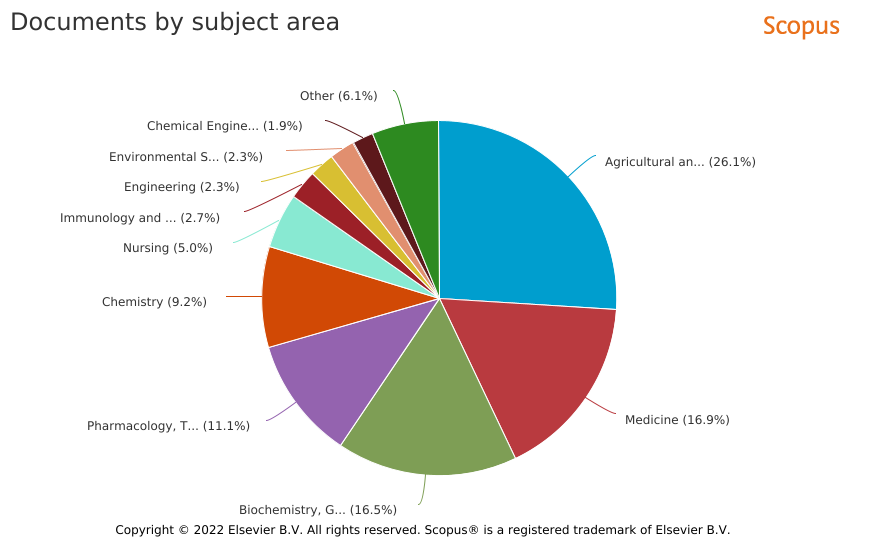

Supplement: Supplementary file 1 [file plants-11-03434-s001.zip › SUPPLEMENTARY_MATERIALS_CARBONE_revised/FIGURE_S1b.png]

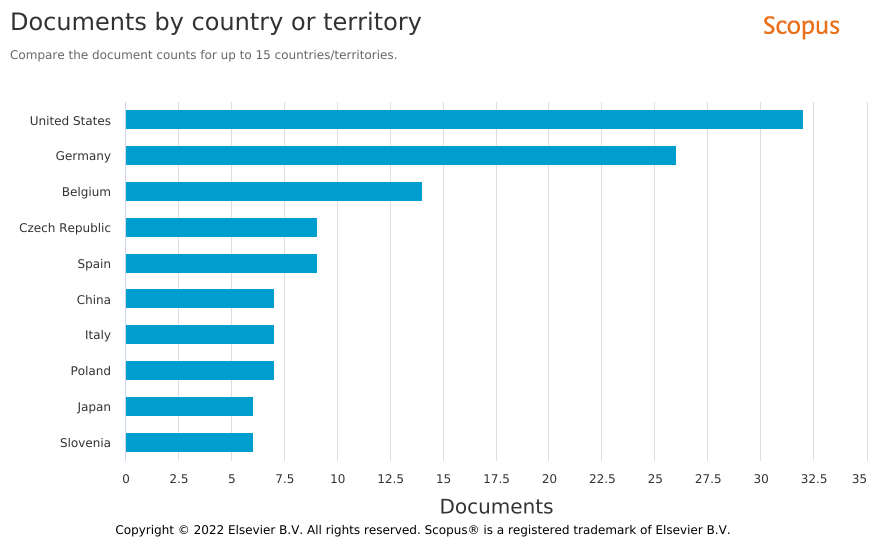

Supplement: Supplementary file 1 [file plants-11-03434-s001.zip › SUPPLEMENTARY_MATERIALS_CARBONE_revised/FIGURE_S1c.png]

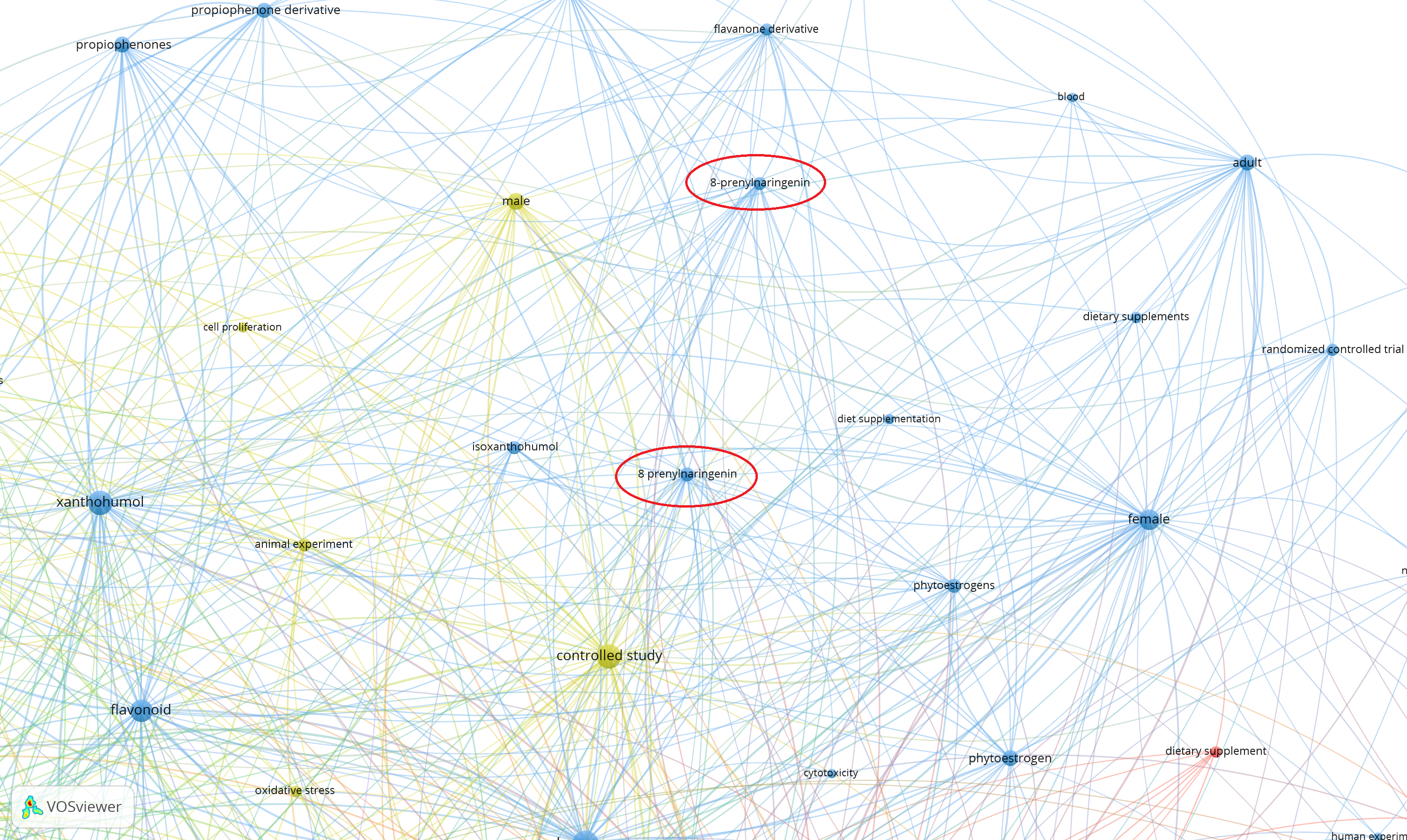

Supplement: Supplementary file 1 [file plants-11-03434-s001.zip › SUPPLEMENTARY_MATERIALS_CARBONE_revised/FIGURE_S2.png]
